# Integrating Genome-based Informatics to Modernize Global Disease Monitoring, Information Sharing, and Response

**DOI:** 10.3201/eid/1811.120453

**Published:** 2012-11

**Authors:** Frank M. Aarestrup, Eric W. Brown, Chris Detter, Peter Gerner-Smidt, Matthew W. Gilmour, Dag Harmsen, Rene S. Hendriksen, Roger Hewson, David L. Heymann, Karin Johansson, Kashef Ijaz, Paul S. Keim, Marion Koopmans, Annelies Kroneman, Danilo Lo Fo Wong, Ole Lund, Daniel Palm, Pathom Sawanpanyalert, Jeremy Sobel, Jørgen Schlundt

**Affiliations:** Technical University of Denmark, Lyngby, Denmark (F.M. Aarestrup, R.S. Hendriksen, O. Lund, J. Schlundt);; US Food and Drug Administration, College Park, Maryland, USA (E.W. Brown);; Los Alamos National Laboratory, Los Alamos, New Mexico, USA (C. Detter);; Centers for Disease Control and Prevention, Atlanta, Georgia, USA (P. Gerner-Smidt, K. Ijaz, J. Sobel);; Public Health Agency of Canada, Winnipeg, Manitoba, Canada (M.W. Gilmour);; University of Münster, Münster, Germany (D. Harmsen);; Health Protection Agency, Salisbury, UK (R. Hewson);; London School of Hygiene and Tropical Medicine, London, UK (R. Hewson, D. Heymann);; Health Protection Agency, London (D. Heymann);; European Center for Disease Control and Prevention, Stockholm, Sweden (K. Johansson, D. Palm);; Northern Arizona University, Flagstaff, Arizona, USA (P.S. Keim);; TGen, Phoenix, Arizona, USA (P.S. Keim);; National Institute for Public Health and the Environment, Bilthoven, the Netherlands (M. Koopmans, A. Kroneman);; Erasmus MC, Rotterdam, the Netherlands (M. Koopmans, A. Kroneman);; World Health Organization, Geneva, Switzerland (D. Lo Fo Wong);; and Ministry of Public Health, Nonthaburi, Thailand (P. Sawanpanyalert)

**Keywords:** genome-based informatics, disease monitoring, information sharing, point-of-care clinical diagnosis, genomic tools, emerging diseases, infectious diseases, outbreaks, bacteria, viruses, parasites, pathogens

## Abstract

The rapid advancement of genome technologies holds great promise for improving the quality and speed of clinical and public health laboratory investigations and for decreasing their cost. The latest generation of genome DNA sequencers can provide highly detailed and robust information on disease-causing microbes, and in the near future these technologies will be suitable for routine use in national, regional, and global public health laboratories. With additional improvements in instrumentation, these next- or third-generation sequencers are likely to replace conventional culture-based and molecular typing methods to provide point-of-care clinical diagnosis and other essential information for quicker and better treatment of patients. Provided there is free-sharing of information by all clinical and public health laboratories, these genomic tools could spawn a global system of linked databases of pathogen genomes that would ensure more efficient detection, prevention, and control of endemic, emerging, and other infectious disease outbreaks worldwide.

Infectious diseases remain a global challenge. Of the 58 million deaths worldwide each year, 15 million (>25%) are the direct result of infectious diseases (*1*). This magnitude, while staggering, is an underestimate because it excludes deaths caused by complications from chronic infections that cause end-stage disease (e.g., cirrhosis or malignancy) or by the lingering consequences of a past infection. Moreover, given trends toward globalization of travel and trade (including food), demographic changes (urbanization, aging population), and the increasing effect of human populations on natural environments, infectious disease challenges will continue to emerge, re-emerge, and cause global threats.

A wide range of specialized culturing and subtyping techniques have traditionally been used to confirm the clinical diagnosis and surveillance of infectious diseases. This approach has been used and validated across almost all infectious disease pathogens, including *Salmonella enterica*, methicillin-resistant *Staphylococcus aureus*, *Mycobacterium tuberculosis*, influenza, and enteroviruses. However, the diagnostic basis for the majority of these methods is to sample a single or small number of defining traits for the target pathogen. Owing to technical limitations, such approaches ignore the vast multitude of traits possessed by pathogens that could otherwise be used for a substantially improved understanding of their identity, virulence, and transmission. Furthermore, current diagnostic methods can require several weeks from collecting a biologic sample to full characterization, and this can lead to delays in determining the source and scope of infectious diseases.

In low-income countries, where the time lag is greatest, delays in diagnosis lead to suboptimal treatment, delayed identification of preventable diseases, and, in some instances, exacerbation of outbreaks. Even in countries with sufficient specialized diagnostic capabilities, the current procedures for identification, subtyping, analysis, and information flow are often slow, leading to delays in disease treatment and detection. Delays are often the greatest when dealing with novel pathogens; such delays give the pathogens the opportunity to spread rapidly, as exemplified by the severe acute respiratory syndrome outbreak in 2003, the emergence of influenza A(H1N1) virus in 2009, and the recent outbreak of enterohemorrhagic *Escherichia coli* in Germany (*2*–*4*).

## Importance of Global Monitoring and Information Sharing

Many human infectious diseases increasingly have a global reach. In addition to the severe acute respiratory syndrome and influenza A(H1N1)pdm09 pandemics, pandemics in poultry of highly-pathogenic avian influenza (H5N1) virus, the intercontinental transmission of *Vibrio cholerae*, and the spread of emergent antimicrobial resistance are exacerbated by global trade and travel. Local infectious disease events have the potential to develop rapidly into international public health emergencies. The specific epidemiologic profile of infectious agents varies because infectious agents can readily transfer between distinct reservoirs. Rapid detection and analysis of and timely public health response to infectious diseases worldwide are crucial for the prevention and global spread of infectious diseases. The World Health Organization International Health Regulations (*5*) encompass these values and provide a useful platform to strengthen the need for global surveillance, monitoring, and reporting.

Specific global networks for identification of infectious agents exist, including the Global Influenza Surveillance Network (www.who.int/csr/disease/influenza/surveillance/en), the PulseNet International partnership for rapidly sharing information on genetic subtypes of enteric pathogens (www.pulsenetinternational.org), and the acute flaccid paralysis surveillance program of the Global Polio Eradication Initiative (www.polioeradication.org/). Notwithstanding the superb achievements of these programs, the time from detection to response is sometimes unacceptably long because of delays in laboratory confirmation.

## New Possibilities

Technological and organizational capacities for the diagnosis and surveillance of infectious diseases require modernization, particularly in developing countries. The current system involves point-of-care laboratories that often do not have supplies or the capacity needed for diagnosis, and they must provide specimens to regional or national laboratories, which may in turn refer to international laboratories for specialized testing and characterization. The result can be failure to correctly diagnose disease and/or failure to detect and prevent local and international spread of emerging or other infectious diseases.

Although the potential for improved disease surveillance through genome sequence sharing in high-throughput laboratory networks has been suggested for some time (*6*), introduction of next-generation sequencing approaches at point-of-care laboratories, coupled with automated data analyses and connectivity to a global genome database, would also enable more rapid diagnosis in the clinical setting in a clinically relevant timeframe. Already, several published examples have shown proof of application and concept (*7*–*12*), and how they have contributed to better patient management; more robust surveillance and risk assessment; and stronger, more focused control strategies, such as optimal use of vaccines.

Global access to a single genomics technology or other cross-compatible technologies will provide diagnostic laboratories at the point-of-care with a standard approach to diagnosis. Using whole genome sequence information for clinical diagnosis and surveillance, linked with clinical and epidemiologic information, would create a common, universal and sustained database that would strengthen clinical management and provide the current and historic data required for more effective risk assessment. It is of great importance to engage stakeholders now, to obtain agreement on a standardized data format (e.g., quality requirements and standardization of reported DNA sequences) that will ensure backward and forward compatibility of genomic information in support of future clinical diagnosis and surveillance.

Standardization, as seen in outbreak detection systems like PulseNet (www.pulsenetinternational.org), is critical for a successful global approach. PulseNet utilizes standardized technology and informatics to acquire and analyze pathogen genotypes in a way that supports full interoperability between all network members. Although the final genome product is entirely standardized and infinitely sharable, the raw DNA sequencing data produced across different genomics platforms are not. This was illustrated during a study of the cholera outbreak in Haiti, in which it was not possible to readily compare data from different studies because of differences in raw data read formats (*10*,*13*). Because of the increasingly rapid pace of genomics, a new set of standards is required for genome sequences (*14*).

The development of new genomic technologies may enable laboratories in developing countries to “leapfrog” and avoid the development of costly laboratory systems similar to those that are being implemented in Organization for Economic Co-operation and Development countries, where separate specialist testing capacities exist for each of the many microbiological families. Such specialization would not be necessary if there was one simple and universally agreed-on approach—such as whole genome sequencing—that could be applied to all infectious agents, including bacteria, viruses, parasites, and even complex polymicrobial infections.

The cost of whole genome sequencing technologies is dropping precipitously, and the most immediate bottlenecks result from a lack of tools critical for Internet-based data analysis. Available software is slow, requires manual interaction, and does not translate complex results into practical information. Software development must become more rapid and permit point-of-care professionals to rapidly evaluate data and interpret results. In the ideal situation, the software should also have the capacity to automatically provide public health warning flags when clusters of disease are occurring in time or space.

Besides whole genome sequencing several other methods and technologies are also becoming more widely used and are under further development. These methods and technologies include use of matrix-assisted laser desorption ionization time-of-flight mass spectrometry (*15*) and different PCR-based methods for direct detection in clinical samples (*16*). Such methods have great advantages in being easy and having a high through-put and low cost. Furthermore, some of the methods may be used directly on the clinical samples, further reducing time and cost. A limitation of the currently available methods is that they do not reliably identify isolates at the subspecies and clonal level, do not give sufficient information on different virulence and resistance genes, and are difficult to standardize in a way that enables global exchange of data. While we believe that whole genome sequencing can provide a single technology solution for the future, it may also be recognized that a combination of methodologies will be the future.

## Future Vision

New sequencing technology will provide the opportunity to create a global system of linked databases for identification and detailed genetic characterization of all microorganisms in clinical (and other) settings. This would enable us to circumvent the need for expansion of existing expensive, cumbersome, and unreliable systems where not fully developed and to partially or wholly replace those in existence. Such a global system would result in a reduction in characterization time (e.g., hours/days instead of weeks). It would also strengthen local, national, and international surveillance of infectious diseases by ensuring more appropriate technology for communicable disease surveillance.

A single technology applicable to different disciplines (e.g., bacteriology, virology, parasitology) and domains (human, food, animal, environment) would facilitate global cross-cutting collaboration and information exchange (integrated surveillance), leading to rapid and coordinated responses to novel and known health threats as they emerge. Such a global system would also provide information across the multitude of microbial variants, and, provided that appropriate clinical and epidemiologic information is collected, would also be useful for studies at the molecular epidemiology level. Technological progress and market forces are currently merging to drive widespread deployment of genomic sequencing technology for routine diagnostic testing in clinical laboratories; thus, at present, there is a window of opportunity to develop a system built on a common data format, ontology, and nomenclature. Informing and engaging relevant stakeholders, relevant funding institutions, the private sector, and all technical constituencies is critical to this effort.

## Implementing a System

The establishment of an efficient global system, or globally interoperable systems, to aggregate, share, mine, analyze, and translate genomic data can only be achieved through effective collaboration across disciplines (e.g., clinicians, veterinarians, microbiologists, epidemiologists, and bioinformaticians), while respecting national and international, legal, and ethical rules and regulations. Such a system could gradually be implemented, starting within a 5–10 year timeframe, and would promote equity in access and use of the current technology worldwide while enabling cost-effective improvements in global health. Several structures could be envisaged, including a global system of data centers at the national or regional level with direct links to major repository databases and evaluation systems.

A prerequisite for such a system is a fundamental shift in the current paradigm of infectious disease detection, which is generally focused on single pathogens. Merging access to laboratory data across traditional disciplines (e.g., virology, bacteriology, parasitology or animal, food, human) would result in more cost-effective diagnosis and surveillance. Capacity-building, especially in developing countries and for presequencing manipulation of samples, is essential, as is the development of simple-to-use bioinformatic tools for rapid analysis of the generated data. Several online and free-to-use tools are under development (*17*–*19*), and more will surely follow.

System transparency must be maintained, and data-sharing rules should be established to govern issues such as capacity for downloads and use of data. Likewise, minimum requirements for assembly of metadata and strain-related data must be agreed upon, and procedures would be required to enable data entry through multiple portals.

Standards should be developed by scientists, health worker, and relevant agencies under the auspices of international health agencies, such as the World Health Organization and the World Organisation for Animal Health, and should be used to support an all-inclusive, non-exclusive database that can be easily accessed by all stakeholders and accommodate all current and future sequencing technologies. Ensuring that new data can be inserted into other databases, both current and historic, could ensure wider acceptance by stakeholders with existing whole genome sequencing platforms.

The system should be capable of providing instant automated reports to each user on demand and of containing the information needed, e.g., molecular typing information, spatial and temporal data, and related information, such as treatment guidelines. To provide the win-win outcome that could strongly promote this endeavor, this function must be useful for clinical and for public health workers. At the same time, diplomacy will be required to engage relevant stakeholders, funding institutions, the private sector, and the technical constituencies and to overcome potential obstacles, such as reluctance of researchers to share data before publication; reluctance of governments and institutions to share data when there are competing interests, such as trade and tourism; legal and ethical issues regarding sharing of information, including patenting and intellectual property; and the need to guarantee confidentiality during public health emergencies and to protect individual patient’s privacy rights. Some of these potential obstacles may be good precautions or required practices and should not necessarily be changed. An expert group addressing the need for global data-sharing for rapid response to public health emergencies (GESTURE; http://ec.europa.eu/eahc/projects/database.html?prjno = 20084153) recently analyzed some of these potential obstacles.

Before implementation, the new methods and systems need to be thoroughly evaluated to ensure their clinical and epidemiologic accuracy and relevance, and, hopefully, their backward compatibility to previous methods of identification and phenotype/genotype characterization. This notwithstanding, there will be a continued need to perform phenotypic testing to ensure that novel genes not previously related with specific phenotypes are detected.

## The Way Forward

The key to success in translating recent developments in sequencing technology into efficient clinical and public health practice is to capture needs and expectations from all involved stakeholders, beginning with clinicians and point-of care laboratories, and public health experts in charge of national surveillance and risk assessment. By linking their needs to those for national, regional, and global public health, a coordinated technology shift would be possible, and a powerful argument for financing could be developed that would be attractive to development agencies and global foundations, providing evidence regarding current costs and estimations of potential cost savings. If coordination cannot be accomplished, the technology shift, which will certainly take place, might not realize its full potential, could have limited practical value, and would not be sustainable.

Early adopters of a coordinated approach are likely to be state-of-the-art laboratories involved in research activities or outbreak investigations. Some of these laboratories are presently running high-resolution sequencing activities in parallel with classical laboratory exercises, and understanding their cost-benefit analysis will be crucial to develop the justification required to convince stakeholders to choose a coordinated path.

In developing countries, current diagnostic methods involve a broad variety of methods and require a lot of training. If the software bottleneck is solved, whole genome sequencing could become a simple one-fits-all tool in a unified system for all infectious diseases. Thus, the system could result in rapid updates of diagnostics in developing countries, much like cellular telephones swept through the same countries much quicker than wired telephones.

Increased global collaboration and information exchange will enable rapid and coordinated response to novel and known health threats. This rapid and coordinated response will have major benefits for local, national, regional, and global public health.
